# Community-based testing of migrants for infectious diseases (COMBAT-ID): observational cohort study measuring the effectiveness of routine testing for infectious diseases among migrants attending primary care

**DOI:** 10.1016/j.eclinm.2025.103253

**Published:** 2025-05-30

**Authors:** Rebecca F. Baggaley, Christopher A. Martin, Helen C. Eborall, Marjan Gohar, Kashif Aziz, Muhammad Fahad, George Hills, Mayur Patel, Iain Stephenson, Pranabashis Haldar, Ibrahim Abubakar, Oliver Toovey, Helena A. White, William Jones, Mark Pierce, Rachna Vyas, Nilesh Sanganee, Caroline Trevithick, Chris Griffiths, Manish Pareek

**Affiliations:** a Development Centre for Population Health, University of Leicester, Leicester, UK; b NIHR Leicester Biomedical Research Centre (BRC), University of Leicester, Leicester, UK; c Department of Population Health Sciences, University of Leicester, Leicester, UK; d Department of Respiratory Sciences, University of Leicester, Leicester, UK; e Institute of Health Informatics, University College London, London, UK; f Department of Infection and HIV Medicine, University Hospitals of Leicester NHS Trust, Leicester, UK; g NIHR Applied Research Collaboration East Midlands, University of Leicester, Leicester, UK; h Usher Institute, College of Medicine and Veterinary Medicine, University of Edinburgh, Edinburgh, UK; i Department of Clinical Microbiology, University Hospitals of Leicester NHS Trust, Leicester, UK; j NHS Leicester Integrated Care Board, Leicester, UK; k Faculty of Population Health Sciences, University College London, London, UK; l Department of Virology, University Hospitals of Leicester NHS Trust, Leicester, UK; m The College of Life Sciences, University of Leicester, Leicester, UK; n Department of Emergency LRI, University Hospitals of Leicester NHS Trust, Leicester, UK; o NHS Leicester, Leicestershire & Rutland Integrated Care Board, UK; p Wolfson Institute of Population Health, Queen Mary University of London, London, UK; q Nuffield Department of Primary Health Care Sciences, Radcliffe Observatory Quarter, University of Oxford, Oxford, UK

**Keywords:** HIV, Viral hepatitis, HBV, HCV, Tuberculosis, Migrant health, Primary care

## Abstract

**Background:**

Migrants are at increased risk of chronic infections and have poorer outcomes, being more likely to present late. Early diagnosis and management can reduce morbidity, mortality and onward infection transmission.

**Methods:**

We evaluated the effectiveness of an integrated approach to screening migrants for exposure to tuberculosis (TB) with an interferon gamma release assay (IGRA) test, HIV, hepatitis B virus (HBV, using hepatitis B surface antigen testing) and hepatitis C virus (HCV, using antibody testing with confirmatory PCR test) infection when patients first registered with general practices (GPs) in Leicester, UK, using test yields (test positivity rates), numbers of new diagnoses and numbers linked to care.

**Findings:**

Of 4004 migrant GP patients referred for testing 2016–2019, test yields were 0.48% (17/3545, 95% CI 0.30–0.77%, HIV), 3.34% (117/3502, 95% CI 2.80–3.99%, HBV), 0.18% (6/3402, 95% CI 0.08–0.38%, HCV) and 19.38% (496/2560, 95% CI 17.89–20.95%, IGRA). Of IGRA-positive patients attending clinic, 7% (31/437) had active TB and 92% (403/437) had latent TB infection. Seventeen (55%) active TB, 397 (99%) latent TB, 71 (61%) HBV, six (35%) HIV and five (83%) HCV infections were new diagnoses. There were high rates of linkage to care for those newly diagnosed. 98% (390/397) of new latent TB patients were offered chemoprophylaxis, of whom 94% (366/390) started treatment and of these, 95% (346/366) completed the course. 100% (6/6), 97% (69/71) and 100% (5/5) of newly HIV-, HBV- and HCV-diagnosed patients attended follow-up, respectively.

**Interpretation:**

This first primary care-based combined infection testing programme for recent migrants found high test yields for latent/active TB, HBV and HIV, substantial numbers of new diagnoses for these infections and excellent linkage to care. To influence UK screening guidelines, its cost-effectiveness and acceptability to other primary care settings must be evaluated.

**Funding:**

10.13039/501100000272NIHR, Gilead Sciences.

**Research in context**

**Evidence before this study**

Certain migrant groups are at increased risk of infectious diseases, including tuberculosis (TB), HIV, hepatitis B virus (HBV) and hepatitis C virus (HCV) infection, and are more likely to be diagnosed late and have poorer outcomes. Earlier diagnosis and management of these infections can reduce morbidity, mortality and onward transmission and is supported by national guidelines.

We searched PubMed for articles published between January 1, 2015, and December 11, 2024, with no language restrictions, using the terms (migra∗) AND ((test∗) OR (screen∗)) AND ((HIV) OR (tuberculosis) OR (hepatitis)). A large majority of identified publications described findings from infection screening programmes across Europe, working in disease silos, focussing on individual diseases at the time of arrival. In particular, blood-borne virus (HIV, HBV and HCV) testing was commonly independent of TB testing programmes. However, there has been a gradual paradigm shift to interest in the feasibility and acceptability of testing for multiple infections and in a variety of settings to remove barriers to testing, with multiple reviews and commentaries advocating multiple infection testing in primary care and qualitative research reporting its acceptability both to healthcare practitioners and to migrants, yet only two pilot studies evaluating its effectiveness in terms of improving infection detection when implemented in primary care, which both adopted a targeted rather than universal approach to migrant testing.

**Added value of this study**

This is the first primary care-based screening programme for universal combined testing for HIV, HBV, HCV and latent plus active TB infection as part of routine care of recent migrants. This programme has already been demonstrated to be acceptable to the target population. We now show that there are high test yields for the interferon gamma release assay (IGRA) test (indicating active or latent TB infection), and for HBV and HIV when testing this migrant population, with relatively low yield for HCV. Substantial proportions of all infections diagnosed represented new diagnoses, demonstrating the utility of this intervention in finding new cases. The high rates of linkage to care for those newly diagnosed and the high treatment completion rates demonstrate the effectiveness of this four-infection testing programme for this migrant population.

**Implications of all the available evidence**

Our findings suggest that universal testing of recent migrants for key infections in the primary care setting is a major tool for identifying new infections and successfully linking patients to care, improving health outcomes for these individuals and the wider community. This approach would complement other successful screening policies such as the UK Emergency Department blood borne virus testing programme, with the common goal of reaching current infectious disease control targets. It has now been demonstrated to be effective and acceptable to patient populations and health care practitioners. Next steps include evaluating the cost-effectiveness of universal testing of migrants in primary care as well as exploring the feasibility of a more targeted, risk-based approach, given the heterogeneities in infection prevalence between migrant groups. In addition, the wide-scale implementation of this approach requires evidence of the generalisability of our findings to other UK settings, and the identification of effective ways to roll out such testing across UK general practices, where time and resources are already overstretched.

## Introduction

Migration is an important determinant of population change in the United Kingdom (UK). The 2021 census found that one in six usual residents of England and Wales were born outside the UK, an increase of 2.5 million since 2011, from 7.5 million (13.4%) to 10 million (16.8%), with India remaining the most common country of birth outside the UK in 2021 (920,000 people, 1.5% of all usual residents).^1^ Large UK urban conurbations in particular attract higher levels of migration and therefore larger overseas-born populations. For example, in Leicester, one of the most ethnically diverse cities in the UK with 59% of the population from ethnic minority groups, approximately 41% of the population is non-UK-born, with nearly 32% born outside Europe according to 2021 census data.^2^

Migrants are a heterogeneous group, characterised by particular language and cultural identities and with specific health needs.^3^ Migrants to the UK from certain regions such as sub-Saharan Africa and Southeast Asia are at increased risk of infection with TB^4^ and blood-borne viruses (BBV) including HIV, hepatitis B and hepatitis C.^5^, ^6^, ^7^ This is due to a combination of factors before, during and after migration, including exposure to infections, poor living conditions and inadequate healthcare access.^8^ Furthermore, there is evidence that migrants are more likely to present late with these infections (e.g., HIV-infection: individuals born overseas are significantly more likely to present with CD4 counts <350 cells/mm^3^^9^), have more aggressive disease processes (TB infection^10^) and may transmit to contacts if undiagnosed.^11^^,^^12^ Therefore early diagnosis and management of these infections can lead to improved outcomes by preventing morbidity, mortality and onward transmission.^13^ This position is supported by several guidelines from the National Institute for Health and Care Excellence (NICE) and other national bodies which advocate screening migrants for active and latent TB,^14^^,^^15^ HIV,^16^, ^17^, ^18^ hepatitis B^6^^,^^19^ and hepatitis C^6^^,^^19^^,^^20^ to contribute towards national prevention targets.^6^^,^^7^^,^^21^^,^^22^ However, the UK Health Security Agency (UKHSA) reported in 2023 that over the previous eight quarters, the proportion of people experiencing delays between reported symptom onset and start of active TB treatment of more than four months was 30%, while the proportion completing treatment within the expected 12-month duration remained static at 80%,^21^ with no evidence of improvement towards action plan targets (UKHSA TB Action Plan Priorities 3.1 and 4.1.1a^23^). In addition, evidence suggests that in low-incidence countries such as England, most active TB cases in migrants result from reactivation of latent TB infection, with the process of migration and its associated stressors potentially acting as a trigger for reactivation in some cases. This highlights the importance of screening and treatment for latent as well as active infection.^24^

Although the Collaborative Tuberculosis Strategy for England recommends identifying latent TB infection in migrants (from countries with TB incidence ≥150/100,000 or sub-Saharan Africa) when they first register with primary care,^25^ screening migrants for individual conditions (i.e. latent TB only) at the time of primary care registration potentially fails to address the range of infectious diseases prevalent among migrant populations. There are common demographic risk factors, such as ethnicity, country of birth and age, that make testing for multiple infectious diseases both rational and feasible. To address the need for more efficient diagnostic processes, guidelines are therefore increasingly recommending a shift from screening for individual diseases to an integrated approach that combines screening for multiple key infectious diseases.^26^ There is therefore a need to explore coordinated testing for a range of infectious diseases in migrant populations at the time of new patient registration with primary care. This would, for the first time, allow us to determine the effectiveness of such a combined infectious diseases migrant health programme in primary care.

Integrated screening for HIV, TB and hepatitis B and C for migrants when first registering with primary care has been shown to be feasible, as well as acceptable to and positively viewed by migrants and healthcare professionals in a novel, routine screening programme conducted in Leicester, UK between 2016 and 2019.^27^ The aim of the current analysis is to evaluate the effectiveness of this programme in terms of test yields (test positivity rates), numbers of new diagnoses and numbers successfully linked into care.

## Methods

### Study setting and Leicester integrated migrant screening programme

This study was conducted in Leicester, UK, one of the most ethnically diverse cities in the UK. The protocol for the screening programme has been described in detail previously.^28^ Briefly, as part of routine care, the national TB screening programme was modified so that newly-arrived migrants registering in primary care and meeting the study programme eligibility criteria (overseas-born individuals aged 16–65 with UK arrival within the previous 5 years from a country with TB incidence ≥150/100,000 or from sub-Saharan Africa or a refugee/asylum seeker) are offered integrated screening for TB infection, HIV, hepatitis B and hepatitis C as part of a new patient health-check. All tested patients are notified of their laboratory test results and individuals testing positive for one or more of the infections are referred to secondary-care infectious diseases specialists for further assessment and management. Registration with primary care was not dependent on migrants accepting testing.

As part of NHS care, general practices in Leicester commenced migrant screening using blood tests for HIV (4th Generation Ab/Ag test), hepatitis B (HBsAg test), hepatitis C (antibody test with confirmatory PCR test for those testing antibody positive, to test for current infection) and TB infection (both latent and active TB infection can be identified through the interferon gamma release assay (IGRA) test used, QuantiFERON Gold in-tube). Only migrants aged 16–35 years were offered the IGRA test, in line with current guidelines.^14^ Eligible patients were identified by staff at the time of general practice (GP) registration/new patient health check (using template prompts) and offered the tests. Patients provided verbal consent for testing. Patients were excluded if they were tourists visiting the UK, were aged <16 years or whose ethnicity was recorded as White British.

Tests were processed by the University Hospitals of Leicester pathology service. This service is the single provider of testing and clinical care in Leicester and covered the entire study patient population. Results were sent to each participant's general practitioner. Migrants testing positive for any of the infectious diseases were referred to secondary care for further management using standard referral pathways.

### Study design, outcomes, measurements and data collection

This observational cohort study evaluated the effectiveness of routine testing for infectious diseases among migrants attending primary care, utilising laboratory and infectious disease clinic records. At the GP registration appointment, patients were asked if they were a recent migrant (entry to the UK <5 years) and the GP then filled in the blood testing template. Variables collected from GP registration data for all migrants registering with primary care and eligible for the screening-service included: practice-level data, demographics (age, sex, self-reported ethnicity), which communicable disease tests were performed (TB, HIV, hepatitis B and hepatitis C) and test results. The primary study outcome was test yield (test positivity rate) for each of the four infection tests. We extracted anonymised testing data from the University Hospitals of Leicester NHS Trust laboratory system, in which recent migrants are identified through a flag on the blood testing template.

After data extraction, identified individuals with age <16 years or ethnicity recorded as White British were excluded. We were not able to obtain data on numbers of tests with indeterminate results or need for repeat testing or further samples, but could identify any test subsequently determined as false positive. Any instances of false positive tests are reported but results were not adjusted for this because the primary study outcomes were test yields.

The clinic notes at the Department of Infection and HIV Medicine, where all individuals testing positive were treated, were reviewed to extract data on secondary outcomes: numbers referred, numbers attending follow-up, and for individuals diagnosed with active TB and latent TB infection, numbers completing treatment and chemoprophylaxis, respectively. For those testing positive for HBV and HCV, treatment eligibility and details of treatment received were extracted. Patients diagnosed with HBV infection were managed as per University Hospitals of Leicester NHS Trust guidelines, which are based on NICE guidance (7).

### Statistical analysis

We report the recorded demographic factors (sex, age and ethnicity) for all tested patients, with test yields for each of the four infections. 95% confidence intervals (95% CIs) for test yield were calculated using the Wilson score method. We used logistic regression to determine the association of these factors with testing positive, presenting adjusted odds ratios (aORs) and 95% CIs. (We did not attempt logistic regression for other outcomes (e.g., numbers referred, numbers attending follow-up, numbers initiating and completing treatment) because of the small sample sizes.) Ethnicity categories were aggregated into fewer categories than the original dataset (Chinese combined with Other Asian, Black Caribbean combined with Other Black, all Mixed categories combined). This was to ensure sufficient numbers per group to allow meaningful comparison of categories in the multivariable logistic regression models. Data were analysed using Stata version 17.

### Ethical approval

The study received ethical approval from the HRA (16/SC/0127) and University of Leicester (UNOLE 0558).

### Role of the funding source

RFB and MPareek were supported by the National Institute for Health and Care Research (NIHR) Applied Research Collaboration East Midlands (ARC EM) and Leicester NIHR Biomedical Research Centre (BRC). RFB and MPareek were supported by NIHR fellowships (RFB: NIHR Advanced Fellowship NIHR302494, MPareek: NIHR Post-Doctoral Fellowship PDF-2015-08-102). The views expressed in this publication are those of the authors and not necessarily those of the NIHR or the Department of Health and Social Care. Gilead Sciences provided an unrestricted grant for the current study but had no other involvement in the study.

## Results

The data presented here represent all test data extracted from the University Hospitals of Leicester laboratory system for patients identified as recent migrants registering with primary care using the blood testing template, with test samples processed from 1st January 2016 (all BBV testing) and 24th May 2016 (IGRA testing) up to 31st December 2019 (all testing). A total of 4004 new migrant GP patients fitting our inclusion criteria were included in the analysis. Table 1 shows the demographic characteristics of included patients, who were relatively young (74% were aged 35 years or less), with 53% female. There was a wide range of self-reported ethnicities, with the majority of Asian ethnicities (42% Indian, 3% Pakistani, 2% Bangladeshi and 16% Other Asian), a further 9% of Black ethnicities, 5% White and 12% Mixed or Other.

**Table 1:** Demographic characteristics of migrant patients registering at Leicester GP clinics tested for infectious diseases as part of the COMBAT-ID programme.

Variable	Description, n (%) Total n = 4004	
Age group^a^, years		
16–25	1299	(32.44%)
26–35	1650	(41.21%)
36–45	595	(14.86%)
46–55	304	(7.59%)
56–65	142	(3.55%)
66+	14	(0.35%)
**Sex**		
Male	1873	(46.78%)
Female	2131	(53.22%)
**Ethnicity**		
Indian	1665	(41.58%)
Pakistani	114	(2.85%)
Bangladeshi	95	(2.37%)
Other Asian	650	(16.23%)
Black African	233	(5.82%)
Black Other	124	(3.10%)
White	200	(5.00%)
Mixed	85	(2.12%)
Other	398	(9.94%)
Not stated	440	(10.99%)

a For 36 participants, age (years) varied between the tests processed (because tests for different infections were processed on different dates). In these cases, the mean age across all tests processed was used.

### Infection testing and prevalence

Table 2 shows that a similar number of tests was carried out for the three BBVs (3545 for HIV, 3502 for HBV, 3402 for HCV), with fewer IGRA tests (testing for active and latent TB infection) conducted (2560) because TB testing guidelines recommend testing for new migrants up to 35 years of age only.^29^ Test yield was 0.48% (17/3545, 95% CI 0.30–0.77%) for HIV, 3.34% (117/3502, 95% CI 2.80–3.99%) for HBV and 0.18% (6/3402, 0.08–0.38%) for HCV, with 19.38% (496/2560, 95% CI 17.89–20.95%) positive on the IGRA test, indicating active or latent TB infection.

**Table 2:** Test yields for HIV, hepatitis B and C and latent/active TB^a^ infection stratified by demographic characteristics.

	HIV		Hepatitis B		Hepatitis C		TB^a^ infection	
	x/n	(%, 95% CI)	x/n	(%, 95% CI)	x/n	(%, 95% CI)	x/n	(%, 95% CI)
**All**	17/3545	(0.48%, 0.30–0.77%)	117/3502	(3.34%, 2.80–3.99%)	6/3402	(0.18%, 0.08–0.38%)	496/2560	(19.38%, 17.89–20.95%)
Age group (years)								
16–25	2/1109	(0.18%, 0.05–0.66%)	18/1095	(1.64%, 1.04–2.58%)	0/1068	(0.00%, 0.00–0.36%)	172/1133	(15.18%, 13.21–17.39%)
26–35	5/1397	(0.36%, 0.15–0.84%)	54/1395	(3.87%, 2.98–5.02%)	2/1347	(0.15%, 0.04–0.54%)	319/1416	(22.53%, 20.43–24.78%)
36–45	5/588	(0.85%, 0.36–1.97%)	28/580	(4.83%, 3.36–6.89%)	1/565	(0.18%, 0.03–1.00%)	2/5	(40.00%, 11.76–76.93%)
46–55	5/299	(1.67%, 0.72–3.85%)	12/286	(4.20%, 2.42–7.19%)	3/280	(1.07%, 0.37–3.10%)	2/3	(66.67%, 20.77–93.85%)
56–65	0/140	(0.00%, 0.00–2.67%)	5/133	(3.76%, 1.62–8.50%)	0/130	(0.00%, 0.00–2.87%)	1/2	(50.00%, 9.45–90.55%)
66+	0/12	(0.00%, 0.00–24.25%)	0/13	(0.00%, 0.00–22.81%)	0/12	(0.00%, 0.00–24.25%)	0/1	(0.00%, 0.00–79.35%)
**Sex**								
Male	9/1718	(0.52%, 0.28–0.99%)	82/1701	(4.82%, 3.90–5.94%)	4/1643	(0.24%, 0.09–0.62%)	269/1071	(25.12%, 22.61–27.80%)
Female	8/1819	(0.44%, 0.22–0.87%)	35/1801	(1.94%, 1.40–2.69%)	2/1759	(0.11%, 0.03–0.41%)	227/1489	(15.25%, 13.51–17.16%)
**Ethnicity**								
Indian	1/1444	(0.07%, 0.01–0.39%)	22/1418	(1.55%, 1.03–2.34%)	3/1392	(0.22%, 0.07–0.63%)	266/1196	(22.24%, 19.97–24.68%)
Pakistani	0/86	(0.00%, 0.00–4.28%)	2/84	(2.38%, 0.66–8.27%)	0/79	(0.00%, 0.00–4.64%)	15/77	(19.48%, 12.18–29.69%)
Bangladeshi	0/77	(0.00%, 0.00–4.75%)	6/77	(7.79%, 3.62–15.98%)	0/76	(0.00%, 0.00–4.81%)	9/63	(14.29%, 7.70–24.97%)
Other Asian (inc Chinese)	1/577	(0.17%, 0.03–0.98%)	26/574	(4.53%, 3.11–6.55%)	1/561	(0.18%, 0.03–1.00%)	46/383	(12.01%, 9.13–15.65%)
Black African	8/211	(3.79%, 1.93–7.30%)	16/210	(7.62%, 4.74–12.02%)	0/196	(0.00%, 0.00–1.92%)	49/140	(35.00%, 27.60–43.21%)
Other Black (inc Caribbean)	4/108	(3.70%, 1.45–9.14%)	6/106	(5.66%, 2.62–11.80%)	0/98	(0.00%, 0.00–3.77%)	14/63	(22.22%, 13.73–33.91%)
White, non-British	0/185	(0.00%, 0.00–2.03%)	13/188	(6.91%, 4.09–11.47%)	0/181	(0.00%, 0.00–2.08%)	17/110	(15.45%, 9.88–23.36%)
Mixed	1/90	(1.11%, 0.20–6.03%)	5/80	(6.25%, 2.70–13.81%)	0/75	(0.00%, 0.00–4.87%)	16/54	(29.63%, 19.14–42.83%)
Other	0/41	(0.00%, 0.00–8.57%)	10/370	(2.70%, 1.47–4.90%)	2/357	(0.56%, 0.15–2.02%)	26/230	(11.30%, 7.83–16.05%)
Not stated	2/403	(0.50%, 0.14–1.79%)	11/395	(2.78%, 1.56–4.92%)	0/387	(0.00%, 0.00–0.98%)	38/244	(15.57%, 11.56–20.65%)

a The IGRA test used identifies both active and latent TB infections.

Table 2 presents test yields stratified by sex, age group and ethnicity using the condensed ethnicity categories used in the multiple logistic regression models. While more tests were performed among migrants in younger age groups, test yields tended to be highest among older age groups (e.g., highest prevalence for HIV and HCV in the 46-55-year group (1.66% and 1.07%, respectively), and for HBV in the 35–45-year group (4.83%)). Despite TB testing guidelines recommending testing for migrants aged 35 years or younger, a few patients in older age groups were also referred for IGRA testing and had high test yields (5/11, 45% 95% CI 21–72% for all age groups 36+ years combined), which may reflect specific referral for testing because of other TB risk factors. IGRA test yield was high in both testing-eligible age groups (15.2% for 16–25-year-olds, 22.5% for 26–35-year-olds).

Test yield was higher among males for all four infections and there was high heterogeneity in yield by ethnicity (Table 2). There was high prevalence of HIV, HBV and IGRA test yield in Black African and Other Black categories (HIV: 3.79% and 3.57%, HBV: 7.62% and 5.66%, IGRA: 35.00% and 22.22%, respectively). HIV prevalence was also relatively high in the Mixed category (1.25%, 1/90), but this represented only one infection; prevalence was low (<0.5%) in all other ethnicity categories. HBV and IGRA test yields were both much higher than for HIV. As well as Black categories, HBV was particularly high among Bangladeshi and White migrants (7.79% and 6.91%, respectively) and those of Mixed ethnicity (6.25%). HCV test yield was very low, with 0.00% yield for all ethnic groups except for Indian (0.22%), Other Asian (0.18%) and Other (0.56%). IGRA test yield ranged from 11.30% in the Other category to 35.00% among Black Africans.

**Fig. 1:**
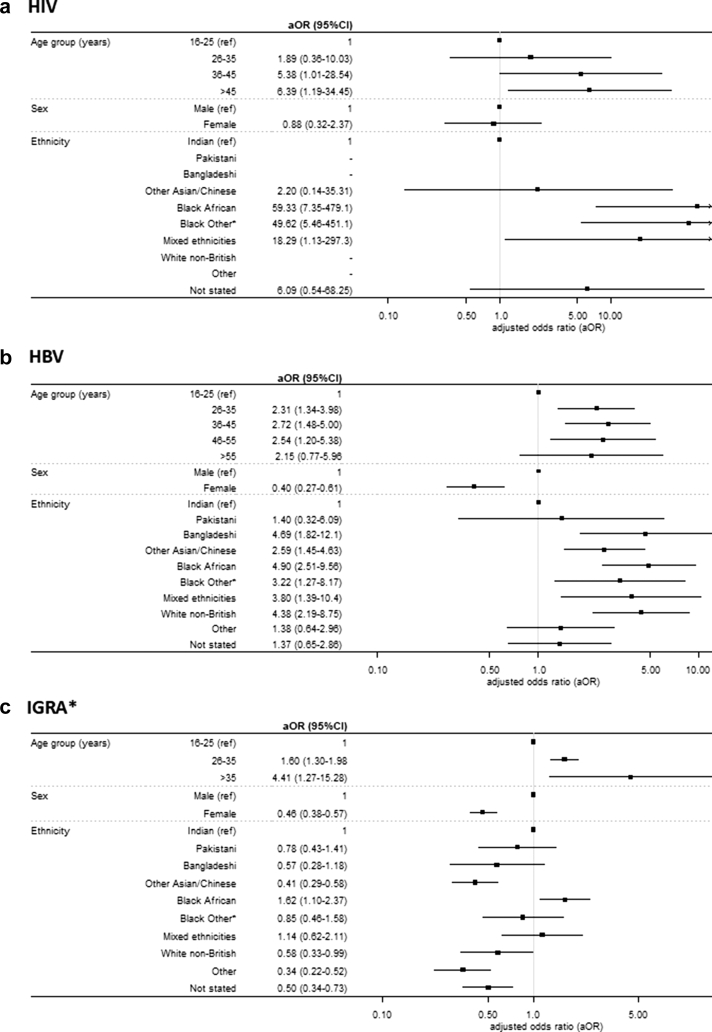
Results of multivariable logistic regression models showing the association of age, sex and ethnicity with testing positive for HIV, HBV and IGRA tests (numbers testing positive for HCV were too low to present a meaningful analysis). Error bars represent adjusted odds ratio 95% confidence intervals. ∗The IGRA test identifies both active and latent TB infections.

Results from the multivariable logistic regression models are presented in Fig. 1 for HIV, HBV and IGRA testing. There were insufficient numbers testing positive for HCV to perform a meaningful analysis and so results are not shown, and similarly for HIV, low numbers testing positive mean that observed differences did not reach significance. After adjusting for all available demographic variables (age, sex, ethnicity), results suggested that the risk of being infected increases with age for all three infections. Women were significantly less likely to test positive for HBV and IGRA tests than men (HBV aOR 0.41 95% CI 0.27–0.61; IGRA aOR 0.46 95% CI 0.38–0.57), but there was no significant difference between sexes for HIV. There were different patterns of test yield by ethnicity for HBV and IGRA tests. While Black Africans were more likely to test positive than Indians (reference group) for both infections (HBV aOR 4.90 95% CI 2.51–9.56; IGRA-positive aOR 1.62 95% CI 1.10–2.37), in general, most ethnic groups had higher HBV test yield than the reference group, but lower IGRA test yield.

### Prevalence of multiple infections

**Fig. 2:**
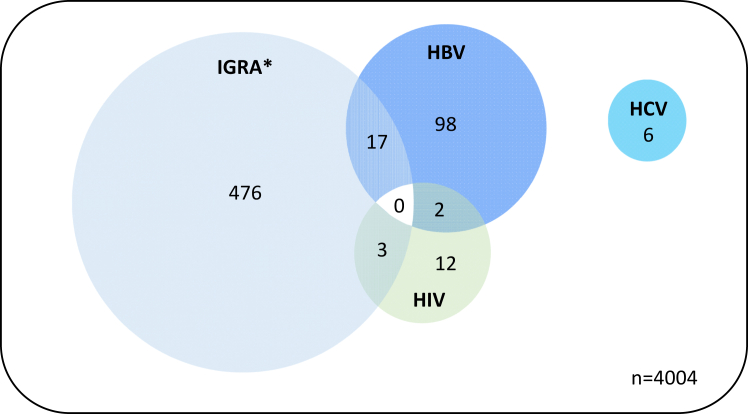
Co-infection prevalence (number of positive tests). Plot not to scale. ∗ The IGRA test used identifies both active and latent TB infections.

Two patients (0.06%) tested HIV-HBV co-positive, three (0.14%) were HIV-IGRA test co-positive and 17 (0.80%) were HBV-IGRA test co-positive (16 had latent TB and one active TB infection) (Fig. 2 and Table S1, Supplementary Material). There were no coinfections with HCV and no multiple infections involving more than two infections.

### New diagnoses and linkage to care

**Fig. 3:**
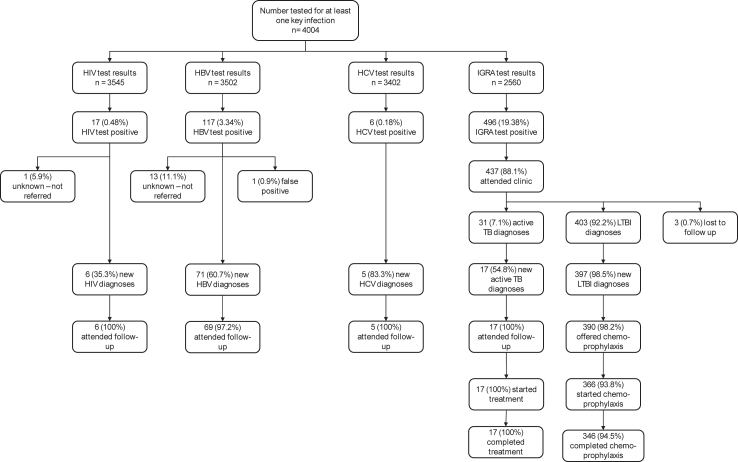
Patient flow diagram: numbers of patients tested as part of the COMBAT-ID screening programme for recent (≤5 years since entry) migrants to the UK by infection type and associated outcomes. Further details are provided in the text and in Figures S2, S3 and S4, Supplementary Material, for patients testing positive for HIV, HBV and TB infection, respectively. LTBI, latent tuberculosis infection.

Of the 17 patients testing positive for HIV infection, 10 (59%) had been previously diagnosed, of whom nine were being treated and continued to attend follow-up (Fig. 3 and Figure S2, Supplementary Material). The other previously diagnosed patient was not undergoing treatment at the time of screening, but as a result of the testing programme, they were offered and accepted treatment and continued to attend follow-up. One patient was not referred for clinic attendance, but no reasons for this had been entered on the clinical system. The remaining six patients (35%) represent new HIV diagnoses as a result of the testing programme. Median first viral load measure for these patients was 76,000 copies/ml (range 673–850,000 copies/ml) and median lowest recorded CD4 count was 165 cells/mm^3^ (range 30–310 cells/mm^3^). All six patients were offered and accepted treatment and continued to attend follow-up.

Of the 117 patients testing positive for HBV infection, one result was recorded as a false positive; this was due to a rare instance of a patient being screened for HBV after recent HBV vaccination (Fig. 3 and Figure S3, Supplementary Material). Thirty-two (37%) patients had been previously diagnosed and 13 (11%) had not been referred for clinic attendance, for reasons unknown. Therefore 71 (61%) patients were referred to clinic, two of whom did not attend. Of the 69 who attended their first clinic appointment (97% of those referred), five were immediately eligible for treatment; two of these were lost to follow-up while the remaining three accepted treatment and continued to attend follow-up appointments. Forty-nine of the 64 clinic attendees ineligible for treatment (77%) continued to attend appointments, of whom 13 (27%) became eligible for treatment during follow-up and 12 (92%) were offered and accepted treatment and continued to attend follow-up appointments.

Of the six patients testing positive for HCV infection, one had been previously diagnosed in 2012 and had not attended for treatment. The five remaining represented new HCV diagnoses (83%), of whom four had undetectable viral load. The one patient with a detectable viral load was treated and achieved sustained virologic response with no recurrence.

Of the 496 patients testing IGRA positive, 37 (7%) were not referred for clinic attendance, for reasons unknown (Fig. 3 and Figure S4, Supplementary Material). Of the 459 referred to clinic, 437 (95%) attended, of whom 31 (7%) were diagnosed with active TB infection and 403 (92%) with latent TB infection. Seventeen (55%) of the active TB infections and 397 (99%) of the latent TB infections were newly diagnosed. For latent TB infection, 390 (98%) were offered chemoprophylaxis; 366 (94%) started treatment, of whom 346 (95%) completed their course.

## Discussion

We report the findings of the first primary care-based screening programme for combined testing for HIV/HBV/HCV/TB as part of routine care in the UK. We found testing yields of 0.48% for HIV, 3.34% for HBV, 0.18% for HCV and 19.38% for IGRA (testing for latent/active TB) for this programme testing recent migrants to the UK in primary care in Leicester city. Importantly, a high proportion of these represented new infection diagnoses for these patients: there were 17 cases of active TB, 397 of latent TB, 71 of HBV, 6 of HIV and 5 of HCV that would have remained unidentified in the absence of this testing programme. These findings demonstrate the public health benefits of this programme (compared to the current situation of ad hoc testing at the discretion of each GP), as earlier diagnosis of these 636 infections will have reduced morbidity, mortality and costs of treating more advanced infections, both to the health system and to the patient, as demonstrated by the high rates of linkage to care that were achieved: for those newly diagnosed, 98% of new latent TB patients were offered chemoprophylaxis, of whom 94% started treatment and of these, 95% completed the course. 100% of newly diagnosed HIV patients, 97% of newly diagnosed HBV patients and 100% of newly diagnosed HCV patients attended follow-up.

HCV prevalence amongst participants in our study was low (0.18%), in agreement with other UK studies^30^, ^31^, ^32^, ^33^ Our findings confirm that UK migrants attending primary care are not a significant risk group for HCV and that current efforts made in the UK to work towards World Health Organization (WHO) elimination targets should target other HCV risk groups and specific migrant groups at higher risk of infection, such as blood transfusion recipients. However, this recommendation is dependent on current HCV prevalence in countries of origin for migrants arriving in the UK. Migrants from high HCV prevalence countries may tend to settle in European countries other than the UK, and so our finding of low HCV among migrants may not be generalisable to other European countries.

In contrast to HCV, we identified high numbers of migrants with HBV infection. Overall prevalence was 3.3%, which is within the wide range of previous estimates for UK migrants (0.3%^33^^,^^34^–3.5% among asylum seekers^35^ and 4.8% reported among unaccompanied asylum-seeking children^36^). This reflects the heterogeneity in migrants being tested, as HBV prevalence varies considerably by migrant type (e.g., country of birth, sex and age^30^^,^^37^). Similarly, we found that prevalence varied substantially by ethnicity and with increased odds of infection for men and among older age groups, which likely reflects patterns of HBV vaccination coverage in countries of origin.

For HIV and TB, we also found differences in risk by age and ethnicity. For IGRA test yield (indicating presence of active or latent TB infection), men had higher odds of testing positive than women, but there was no meaningful difference between the sexes for HIV test yield. This contrast in infection risk by sex for the different infections should be explored further.

The 0.48% HIV prevalence estimate among migrants in this study is somewhat higher than estimates from other large-scale studies, including 0.26% among migrants attending Genitourinary Medicine (GUM) clinics,^38^ 0.19% among unaccompanied asylum-seeking children^31^ and 0.36% among refugees.^30^ It is not surprising for our sample of adults to have higher HIV prevalence than asylum-seeking children, and while GUM clinic attendees will represent a high HIV risk population, only 4.4% of that sample were from Africa, while 9% of migrants in our sample were Black ethnicity and therefore at higher risk of infection. There remain many other differences between these studies (data collection dates, regions of UK) which make comparisons difficult. NICE testing guidelines define high prevalence and extremely high prevalence areas of England as 0.2–0.5% and 0.5% or more respectively, and recommend offering HIV testing to everyone registering with a GP practice in such areas.^16^ Therefore the HIV test yield found in this study justifies HIV testing for this target group. The high viral loads and low CD4 counts of the newly-HIV diagnosed patients further justifies this testing approach. Opt-out BBV testing in Emergency Departments, currently expanding across the UK and now approved to commence in Leicester in November 2024, has demonstrated another important approach for identifying people with previously undiagnosed infections. An interim evaluation of the first 12 months of the programme found test yields of 1.1% for HBV, 0.9% for HIV and 0.2% for HCV in five sites in London,^39^ where HBV prevalence in particular was found to be high (compared to a general population estimate of 0.45%).

Our study found high IGRA test yields across men and women and all age groups and ethnicities. This demonstrates the importance of enforcing current UK TB testing guidelines,^14^ as high yields for 16–35-year-olds on IGRA test were demonstrated (15.2% testing positive among 16–25-year-olds, 22.5% among 26–35-year-olds). A small number of migrant patients in older age groups were also tested, with the flag on the blood testing template indicating their migrant status, but the sample size is small (5/11, 45% 95% CI 21–72%). While it is likely that the high prevalence is due to referral testing for additional risk factors for TB infection beyond migrant status, we are unable to verify this, given the data available. Our findings support the use of the current testing guidelines^14^ and the UK TB action plan, which aims to improve the detection and treatment of latent TB infection in new migrants.^15^ TB prevention and control are particularly important now, given that TB notifications are increasing in the UK.^4^

Provisional UK data suggest an increase in TB notifications 2022–2023 compared with the previous two years, up to a level seen before the COVID-19 pandemic,^4^ with similar patterns demonstrated in many other countries.^40^ This increase is moving the UK further away from achieving the WHO elimination targets for 2035 (reduce TB deaths by 95% and TB incidence by 90% compared to 2015).^41^ The 19.38% IGRA test yield observed in our study is from tests taken before the COVID-19 pandemic and there are so far insufficient data from published studies of prevalence among migrants from the pandemic onwards to demonstrate increased prevalence in this group.^31^^,^^42^ We do know that the proportion of UK TB notifications accounted for by people born outside the UK has been steadily rising for a number of years.^43^ However, the increase in TB in 2023 was seen in both UK-born and non-UK-born populations in England,^43^ suggesting that the increase is not solely due to infection importation through migration. Travel to high TB prevalence regions by both UK-born and non-UK-born UK residents, and transmission within the UK, are likely to be contributing factors. The UKHSA is currently working with partners to investigate these potential causes.^43^

There are a number of limitations to this analysis, including data recording issues, which are inevitable when evaluating programmes of this kind which involve integrating interventions within routine care. These have led to some incomplete data and potentially some data misclassification; however, there has been rigorous data cleaning and validation prior to analysis to minimise such issues. These data from routine care are therefore also restricted in terms of variables recorded and so we were limited in our evaluation of heterogeneities within the sampled population, only exploring differences in test yield by age, sex and ethnicity (we would have liked to explore differences in prevalence by country of birth, but this information was poorly recorded). The limited sample size for some subgroups based on these demographic characteristics led to us combining some subgroups in our analysis (e.g., combining Chinese with Other Asian, and Black African with Black Caribbean, categories). We chose the aggregate subgroups to achieve balance between sufficient sample sizes for meaningful analysis and the heterogeneity within subgroups, which we acknowledge cannot be perfect, given the very large number of ethnic groups in our sample, and may cloud differences within groups. Test yield may be a slight underestimate of true test yield for all new migrants registering for primary care, if registering undocumented migrants do not feel confident to reveal their migrant status to their GP, and if their infection prevalence is higher than for migrants willing to disclose their status (which may be the case for a number of reasons, including infection acquisition during travel to the UK). Our test yields represent important estimates of infection prevalence amongst migrant populations to the UK; however, there may be some bias as migrants registering with a GP may not be representative of all migrant types, particularly undocumented migrants. This highlights the importance of offering multiple opportunities to test, including primary care, Emergency Department testing and third sector initiatives. Our study has demonstrated the feasibility, acceptability and effectiveness of this testing programme, with relatively high yields for HIV, active/latent TB and HBV. Therefore its adoption in other parts of the UK, as similar programmes are implemented, will provide real-time effectiveness data.

There have been two pilot studies of multiple infection testing in primary care; a large cluster randomised controlled trial in Spain^44^ and a smaller feasibility study in the UK,^45^ which in contrast to COMBAT-ID, both adopted a targeted rather than universal approach to migrant testing, based on risk factors for infection. These studies reported improved infection detection, but a formal comparison with a universal approach would highlight the trade-off between costs saved and new diagnoses missed, and further research is required concerning the risk of re-stigmatising infection testing by emphasising that testing is prioritised based on risk.

We have found that this integrated approach to screening recent UK migrants for key infections in primary care yields large numbers of infections that would otherwise have remained undiagnosed, particularly for HBV and latent/active TB infection, with successful linkage to care for new patients. The testing programme has been demonstrated to be feasible, positively viewed by, and acceptable to migrants and healthcare professionals.^27^ Our findings are important for shaping testing policy and informing clinical practices in real-world settings. We must now evaluate its cost-effectiveness, the feasibility of rolling out such testing when GP clinics’ time and resources are already overstretched, and its generalisability to other UK settings, where prevalence of infections may differ from the Leicester context and patients may be managed across GUM and Respiratory departments rather than in a single Infectious Diseases Department. The cost-effectiveness of offering HCV testing within this package for this population must be evaluated, given the lower test yield; however, there are likely to be economies of scale with combined testing of all three BBVs (HIV/HBV/HCV). Furthermore, there is appeal to migrants and healthcare professionals of offering multiple tests as a unified healthcare package. We must also explore redundancies in this testing approach, as there was great heterogeneity in test yields by demographic characteristics (age, sex, ethnicity), suggesting that a more nuanced, targeted approach to testing based on infection risk may reduce costs both to the health system and patients, as well as reducing the anxiety that may be experienced by patients undergoing unnecessary testing. The findings from the current study represent an important step in defining the best approach for combined testing of migrants, as further programmes of this type are developed and implemented in an increasing number of geographical settings.

## Contributors

MPareek, HE, IA and CG conceived the study idea. MPareek and CM accessed and verified the data used in this study. MG, KA, MF and WJ performed additional data extraction. CM and RFB conducted the data analysis under the guidance of MPareek. MPareek, MPatel and IS set up and ran the migrant testing service. RFB and CM wrote the first draft of the manuscript with input MPareek, GH, OT and HW. All authors reviewed the manuscript and approved the final version prior to submission; they all accept responsibility to submit for publication.

## Data sharing statement

The participants of this study did not give explicit written consent for their data to be shared publicly, so due to the sensitive nature of the research, supporting data are not available.

## Declaration of interests

MPareek reports grants from Gilead for the current work, Sanofi, and Moderna outside the current work, and has received consulting fees from QIAGEN and Gilead. IS reports payment from Gilead (speaker fee for lecture on HIV epidemiology) and support for attending meetings (online registration for 2 international HIV meetings AIDS 2024 and Glasgow HIV conference 2024) from ViV. We report no other relationships or activities that could appear to have influenced the submitted work.
