# Supplementary material for: Community-based testing of migrants for infectious diseases (COMBAT-ID): observational cohort study measuring the effectiveness of routine testing for infectious diseases among migrants attending primary care
Source: eClinicalMedicine. 2025 May 30;84:103253. doi: 10.1016/j.eclinm.2025.103253 (PMC12273839; doi:10.1016/j.eclinm.2025.103253)
Supplement: Supplementary Material [file mmc1.docx]

**SUPPLEMENTARY MATERIAL**

**Community-based testing of migrants for infectious diseases (COMBAT-ID): an observational cohort study evaluating the effectiveness of routine testing for infectious diseases among migrants attending primary care**

***Baggaley, Martin et al.***

[**FIGURE S1** HIV, HBV, HCV and latent/active TB* test yields for migrants stratified by ethnicity (please note different y axis scales). Error bars represent 95% confidence intervals. * The IGRA test used identifies both active and latent TB infections. 2](#_Toc183523241)

[**FIGURE S2** Patient flow diagram for patients testing positive for HIV. 3](#_Toc183523242)

[**FIGURE S3** Patient flow diagram for patients testing positive for HBV. 4](#_Toc183523243)

[**FIGURE S4** Patient flow diagram for patients testing IGRA positive (identifying both active and latent TB infection). 5](#_Toc183523244)

[**TABLE S1** Co-infection prevalence amongst migrants (no subjects tested positive for more than two infections; denominators represent all migrants who were tested for both infections). 5](#_Toc183523245)

# **FIGURE S1** HIV, HBV, HCV and latent/active TB* test yields for migrants stratified by ethnicity (please note different y axis scales). Error bars represent 95% confidence intervals. * The IGRA test used identifies both active and latent TB infections.

# **FIGURE S2** Patient flow diagram for patients testing positive for HIV.


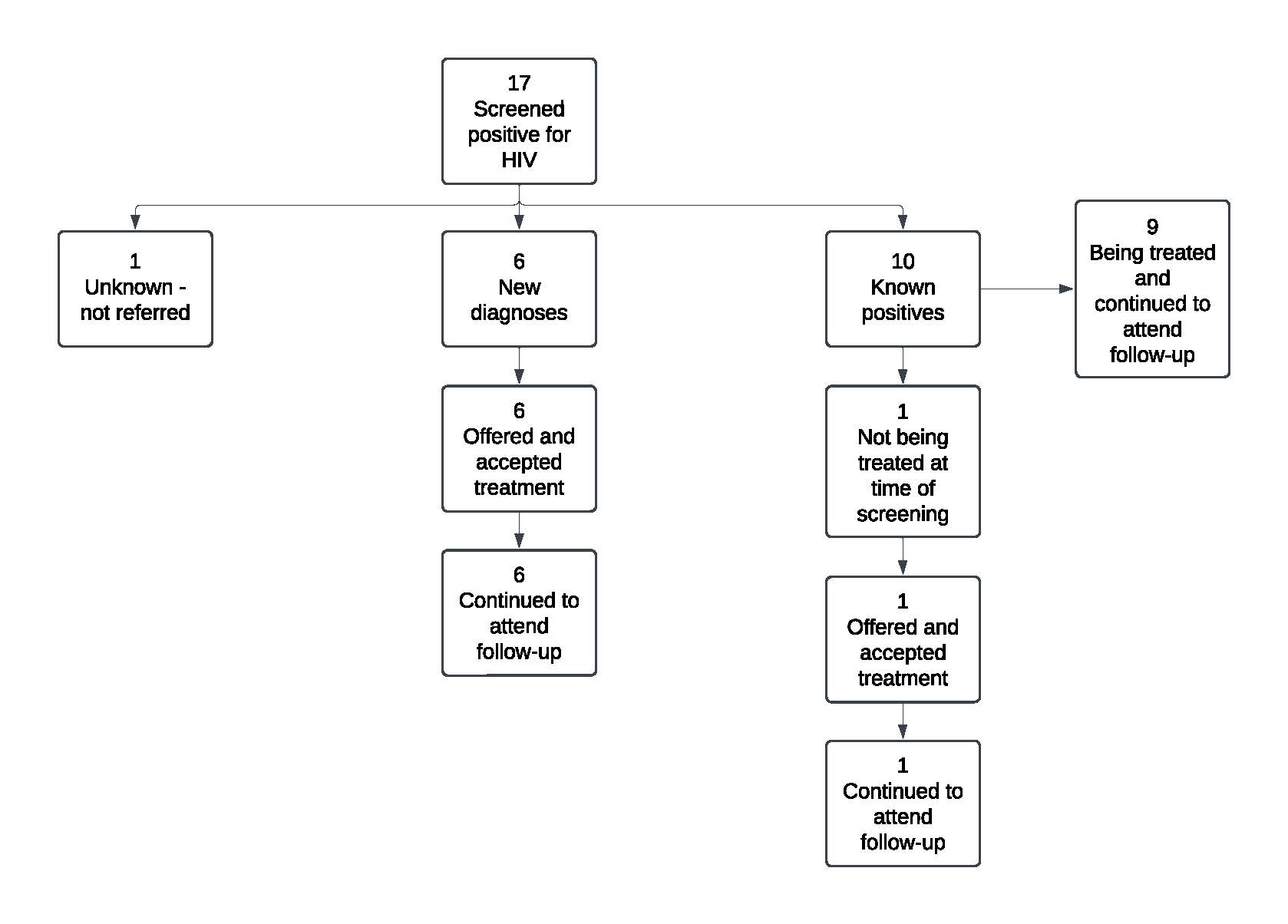


## **FIGURE S3** Patient flow diagram for patients testing positive for HBV. DNA – Did Not Attend.


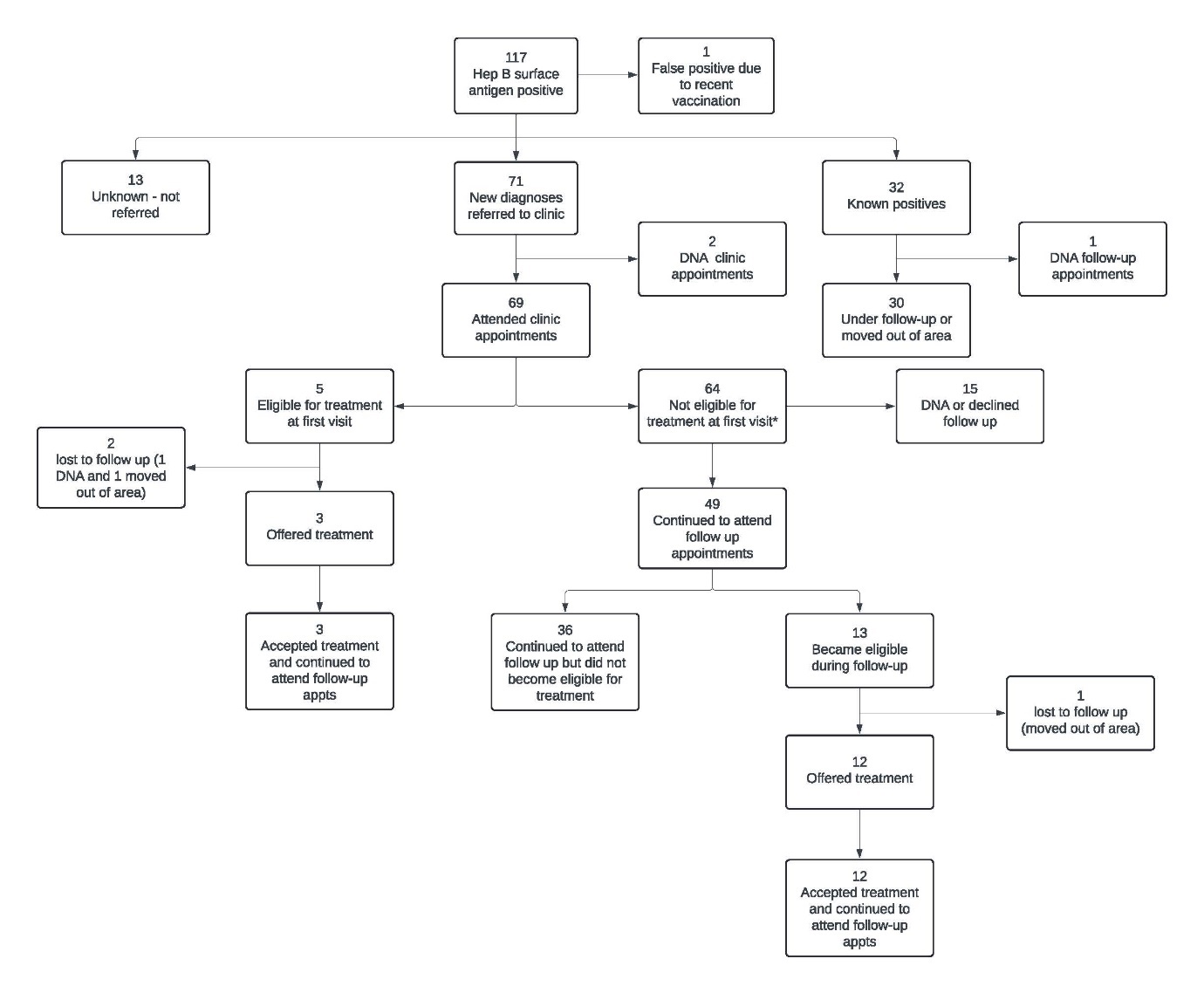


# **FIGURE S4** Patient flow diagram for patients testing IGRA positive (identifying both active and latent TB infection).


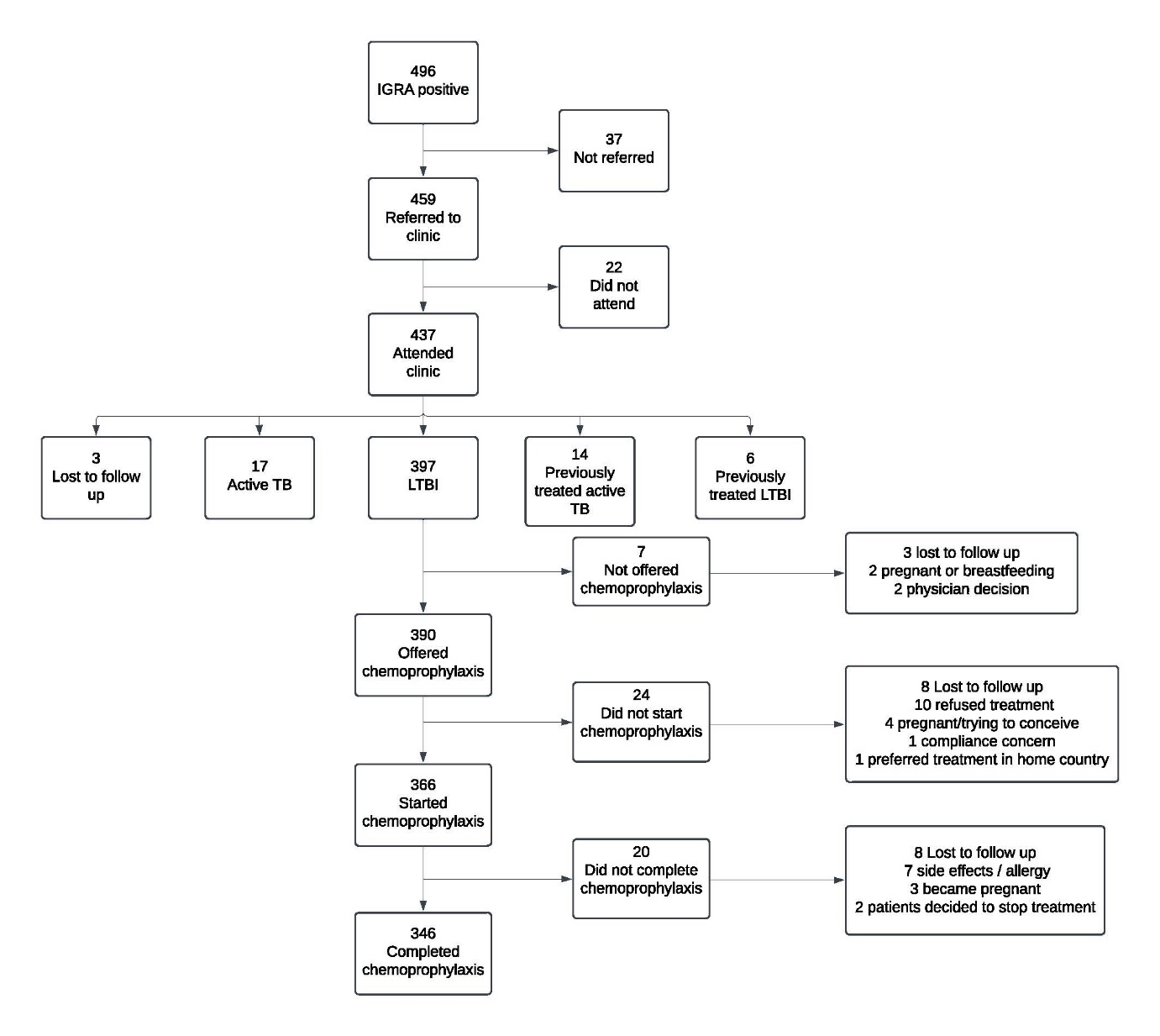


#

# **TABLE S1** Co-infection prevalence amongst migrants (no subjects tested positive for more than two infections; denominators represent all migrants who were tested for both infections).

|  | **HIV** | | **HBV** | | **HCV** | |
| --- | --- | --- | --- | --- | --- | --- |
|  | **x/n** | **(%)** | **x/n** | **(%)** | **x/n** | **(%)** |
| **IGRA*** | 3/2128 | (0.14%) | 17/2124 | (0.80%) | 0/2065 | (0.0%) |
| **HCV** | 0/3387 | (0.0%) | 0/3398 | (0.0%) |  |  |
| **HBV** | 2/3475 | (0.06%) |  |  |  |  |

* The IGRA test identifies both active and latent TB infections.
